# Molecular dynamics of plant-virus interactions: unravelling the dual role of ubiquitin proteasome system

**DOI:** 10.1007/s44154-024-00210-9

**Published:** 2025-02-10

**Authors:** Veerendra Sharma, Ragunathan Devendran, Manish Kumar, Ramgopal Prajapati, Ritesh Kumar, Ved Prakash

**Affiliations:** 1 Department of Plant Pathology, Kansas State University, Manhattan, KS USA; 2 Charles Tanford Protein Center, Martin-Luther University, Halle, Germany; 3 School of Life Sciences, Jawaharlal Nehru University, New Delhi, India; 4 Boyce Thompson Institute, Ithaca, NY USA

**Keywords:** Ubiquitin proteasome system, Plant viruses, Plant defense, Ubiquitination

## Abstract

Plants response to various biotic and abiotic factors requires not only the de novo synthesis of proteins and enzymes but also their precise and timely degradation. The latter is achieved through protein degradation machinery such as the ubiquitin proteasome pathway (UPS). The UPS plays a central role in maintaining cellular physiology and orchestrating plant response to stresses responses. The UPS regulates all stages of defense response from pathogen perception to mounting defense response, this make the UPS a suitable candidate for host manipulation. Viruses are obligatory intracellular pathogens and master of manipulating host defense machinery for successful infection and spread. Several reports suggest a dynamic interaction between the host UPS machinery and viruses. This review focuses on our current understanding of the involvement of UPS in defense against plant viruses and how viruses have evolved mechanisms to counter and exploit UPS machinery for their advantage.

## Introduction

Human civilization is sustained by plants which are the sources of food and many essential non-food products, such as medicines, wood, textiles, rubber, and industrial chemicals. Throughout the life cycle, beginning from seed germination to senescence and death and defense against abiotic and biotic stresses, plants undergo several processes that involve coordinated action of different cellular machinery and require a high degree of proteome plasticity (Orosa et al. 2020). These processes require the timely synthesis and turnover of regulatory proteins when their role is no longer necessary. This protein turnover is governed by highly sophisticated protein degradation pathways known as the Ubiquitin–proteasome systems (UPS) (Sadanandom et al. 2012) and autophagy (Li and Vierstra 2012; Marshall and Vierstra 2018). The autophagy pathway is responsible for degradation and recycling of cytoplasmic components, including proteins, damaged nuclear fragments, dysfunctional complexes, and even whole organelles whereas the UPS is specifically involved in the turnover of proteins (Hua and Vierstra 2011; Marshall and Vierstra 2018). The UPS is highly conserved among eukaryotes, and involves an intricate array of enzymes and enzyme complexes that attach ubiquitin moieties to target proteins, degrade the ubiquitylated protein and recycle the ubiquitin moieties (Vierstra 2009).

Being sessile in nature, plants are constantly attacked by various pathogens including viruses. During evolution, plants have evolved sophisticated mechanisms to perceive the presence of pathogen-specific signatures, and this perception activates different downstream defense signaling pathways (Jones and Dangl 2006). Viruses are tiny, obligate parasites with a small genome size and encodes a few multifunctional proteins. Viral proteins perform dual functions of hijacking the host’s cellular machinery to support viral replication and spread and simultaneously neutralizing host defense system. This tug-of-war between the host and viruses involves complex multilayered dynamic interactions, and the UPS system plays a crucial regulatory role in these processes by coordinating host protein turnover and targeting viral proteins for degradation. This attribute makes the UPS a potent target of viral pathogens to neutralize theUPS mediated defense.

There is a considerable amount of research evidence available on various host defense pathways such as RNAi, hormones-mediated defense, R-gene mediated defense in virus resistance, but the role of UPS in defense against viral pathogens and in viral pathogenesis have started to unfold recently with evidence suggesting both the antiviral and proviral role of host UPS machinery. This review discusses the progress made in the past two decades to unfold the role of UPS in virus resistance and how viruses have evolved mechanisms to counter UPS-mediated host defense and even utilize UPS components to neutralize host defense.

## The plant UPS system

A proteome represents the complete set of proteins expressed by a plant during its life cycle. The plant proteome remains dynamic throughout the life cycle and requires biosynthesis of new proteins and degradation of proteins whose biological function is not required. This proteome plasticity is governed by the UPS which enables plants to alter their proteome in response to developmental and environmental cues (Smalle et al. 2003; Kurepa et al. 2008; Orosa et al. 2020).

The UPS mediated degradation of a target protein begins with covalent attachment of multiple ubiquitin protein subunits, a highly conserved protein among eukaryotes, a target protein followed by its degradation through 26S proteasome (Callis et al. 1989; Doroodian and Hua 2021). Ubiquitination of a protein is a multistep process that involves sequential actions of ubiquitin-activating (E1), ubiquitin-conjugating (E2), and ubiquitin-ligase (E3) enzymes (Vierstra 2009). In the first step, an E1 activates the ubiquitin moiety by forming a high-energy thioester bond; this activated ubiquitin moiety is then transferred to a cysteine residue of E2. In the final step, the E2 partners with an E3 and transfers ubiquitin to a lysine residue of the target substrate (Vierstra 2009; Callis 2014) for in depth details, see (Callis 2014). This process is repeated multiple times to obtain polyubiquitinated target protein. The components of the UPS show hierarchy in that eukaryote genomes encode for one or two E1, 10 s of E2 and 100 s of E3. The first two enzymes of this enzymatic cascade are highly conserved in eukaryotes with only two E1 protein and 37 predicted E2 proteins are encoded by *Arabidopsis* (Downes and Vierstra 2005; Dielen et al. 2010; Callis 2014). On the other hand *Arabidopsis* genome encodes over 1300 E3 ligases (Downes and Vierstra 2005; Dielen et al. 2010). The abundance and diversity of E3 ligases compared to E1 and E2 shows their role in a wide variety of physiological process including biotic stress response. For further in depth detail on E3 ligases please see (Mazzucotelli et al. 2006; Chen and Hellmann 2013).

The polyubiquitinated proteins become the substrate of 26S proteasome which is highly conserved in eukaryotes including plants. The 26S proteasome is composed of two subunits: the 19S regulatory particle (RP) and the 20S core particle (CP) (Voges et al. 1999; Yang et al. 2004; Bard et al. 2018). The 19S RP is further composed of two subcomplexes: the base and the lid. The base subunit is composed of six distinct ATPase subunits and performs translocation and unfolding of proteins using the energy from ATP hydrolysis, while the lid function as a scaffold for targeted protein engagement and deubiquitination. The 20S CP has a barrel-like structure composed of two sets of α and β rings with distinct peptidase sites. This barrel forms a proteolytic core where the degradation of target proteins occurs (Vierstra 2009; Dielen et al. 2010; Stone 2014).

There is a considerable amount of research evidence available on various host defense pathways such as RNAi, hormones-mediated defense, R-gene mediated defense in virus resistance, but the role of UPS in defense against viral pathogens and in viral pathogenesis have started to unfold recently with evidence suggesting both the antiviral and proviral role of host UPS machinery. This review discusses the progress made in the past two decades to unfold the role of UPS in virus resistance and how viruses have evolved mechanisms to counter UPS-mediated host defense and even utilize UPS components to neutralize host defense.

## Antiviral roles of the UPS

**Fig. 1:**
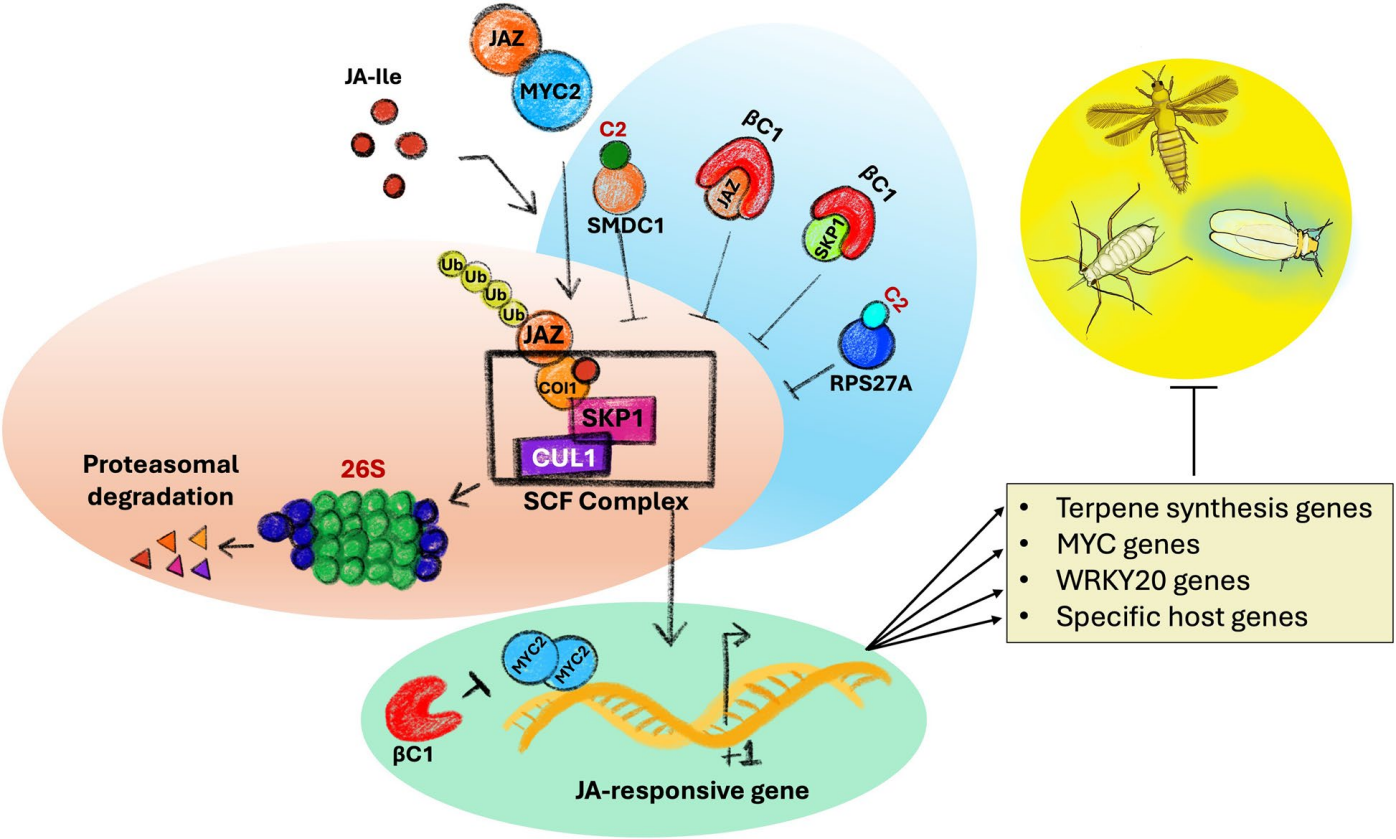
A diagram illustrating the viral proteins involved in modulating proteasomal pathway complex. Viral factors such as βC1 and C2 (begomovirus components) block the ubiquitin pathway and promote viral infection. Consequently, phytohormone signaling gets altered, especially JA signaling, which influences the plant's defense dynamics towards the vector. JAZ = JASMONATE ZIM DOMAIN, MYC2 = Myelocytomatosis transcription factors 2, JA-Ile = Jasmonic acid-isoleucine conjugate, Ub = Ubiquitination factors, SKP1 = S-phase kinase associated protein 1, CUL1 = Cullin1, and RPS27A = ubiquitin-40S ribosomal protein S27a

UPS machinery is highly conserved in eukaryotes and plays a vital role in protein turnover, which is central to the normal functioning of the cells (Callis and Vierstra 1990; Bachmair et al. 1990). To successfully establish an infection, virus-encoded proteins interact with several host proteins and hijack the host’s cellular machinery to create a favorable environment for virus replication, translation, and movement. This multipoint interaction makes viral proteins a suitable target for UPS-mediated degradation for defense against viral pathogens (Fig. 1). Earlier pieces of evidence on the role of the ubiquitin pathway in viral defense come from the work of (Dunigan et al. 1988; Becker et al. 1993; Reichel and Beachy 2000; Jockusch and Wiegand 2003; Takizawa et al. 2005).

Initially, the Ubiquitin pathway was identified as an essential component in N-gene-mediated resistance against a tobamovirus, tobacco mosaic virus (TMV) (Liu et al. 2002). Several viral proteins were identified as the target of the UPS-mediated degradation. The movement protein of a Tymovirus, turnip yellow mosaic virus (TYMV) was identified as a substrate for ubiquitination which undergoes selective proteolysis by UPS machinery (Drugeon and Jupin 2002). TYMV RdRp, when expressed in insect cells, is phosphorylated by the cellular phosphorylase in the putative PEST (Proline, Glutamate, Serine, and Threonine) sequence. Post-phosphorylation ubiquitination of TYMV RdRp in the insect cells suggests that TYMV RdRp is a target of UPS machinery for degradation (Héricourt et al. 2000). Further, site directed mutagenesis of TYMV RdRp in PEST sequence and lysine residues led to the stabilization of Camborde and coworkers provided further evidence for the involvement of UPS in the degradation of TYMV RdRp and regulation of TYMV replication through site-directed mutagenesis of PEST sequence and lysine (Lys) residues. Mutations in the PEST sequence and Lys residues led to the stabilization of RdRp (Camborde et al. 2010). These results suggest that the UPS pathway regulates TYMV replication through the degradation of RdRp. Tombusvirus, tomato bushy stunt virus, (TBSV) encoded P33, a replication cofactor, which functions as an RNA chaperon, and p92pol, which is an RdRp, function together in TBSV replication. In yeast, these proteins interact with Rsp5p, which belongs to the Nedd4 ubiquitin ligase family. Interaction of Rsp5p, with P33 and p92pol, causes selective degradation of p92pol. This selective degradation of TBSV p92pol was independent of the HECT domain of Rsp5p, responsible for ubiquitination. Thus, suggesting that Rsp5p interaction with p33 and p92 rather than ubiquitination is responsible for the inhibition of TBSV replication. Further, it was shown that downregulation of Rsp5p led to higher replication of TBSV repRNA while overexpression of Rsp5p inhibited the accumulation of TBSV repRNA in yeast (Barajas and Nagy 2010; Qin et al. 2012). 

In transgenic *Arabidopsis* plants expressing a GFP fused movement protein (MP17) of a Polerovirus, potato leafroll virus (PLRV), MP17 localizes to secondary branched plasmodesmata (PD) in the source tissue but not to simple PD in the sink tissues. Unraveling the intracellular transport of MP17 using inhibitors of different components of the intracellular transport system indicated that treatment of transgenic plants with a proteasome inhibitor, clasto-lactacystin ß-lactone (CLL) led to the aggregation of MP17 in aggresome-like structures (Vogel et al. 2007). These results suggest that the 26S proteasome system targets the MP17 protein in the sink tissue. The Potexvirus, potato virus X (PVX) TGBp3 movement protein is a target of the ER-associated protein degradation (ERAD) pathway (Ju et al. 2008). Treatment with the proteasome inhibitor MG132 delayed the degradation of GFP-tagged TGBp3 and TGBp3 mutants, indicating that the proteasome degradation pathway controls the accumulation of wild-type and mutant TGBp3: GFP during PVX infection (Ju et al. 2008).

Viral suppressors of RNA silencing (VSRs) are specialized proteins that block antiviral RNA silencing machinery at various stages, and UPS-mediated degradation of VSRs could be a key strategy for developing resistance against plant viruses. This strategy is utilized in rice and *Nicotiana benthamiana* against Tenuivirus, rice stripe virus (RSV). Ubiquitin-like protein 5 (UBL5) from rice and *N. benthamiana* targets silencing suppressor P3 protein through the 26S proteasome pathway. Silencing of NbUBL5 promotes infection of RSV, while over-expression of UBL5 from rice and tobacco confers resistance. Further, UBL5 was found to degrade P3 through ubiquitin receptors such as RPN10 and RPN13, and silencing of either RPN10 or RPN13 abolished the ubiquitin-mediated degradation of P3 (Chen et al. 2020a).

E3 ubiquitin ligases are flexible and highly diverse regulators of the UPS pathway. E3 ligases form the core of ubiquitin-mediated defense and act by attaching ubiquitin moieties to the target protein (Chen and Hellmann 2013; Kumar et al. 2022). In *Nicotiana benthamiana,* Ubiquitin E3 ligase containing RING domain 1 (NbUbE3R1) restricts bamboo mosaic virus (BaMV) replication through binding with BaMV replicase protein. Knockdown of NbUbE3R1 via tobacco rattle virus (TRV) mediated virus induced gene silencing (VIGS) enhanced BaMV replication, while over-expression of *NbUbE3R1* and its derivatives restrict the accumulation of BaMV (Chen et al. 2019). Beta satellites associated with monopartite begomoviruses encode for βC1 protein which function as a pathogenicity factor (Briddon et al. 2003; Kumari et al. 2011). Due to their involvement in symptom induction and neutralizing host defense, βC1 protein becomes a target of the UPS system. An E3 ligase RING-finger protein from tobacco termed as NtRFP1 interacts with a geminivirus, tomato yellow leaf curl virus (TYLCV) encoded βC1 protein, which is a pathogenicity factor. Further investigation revealed that TYLCV-βC1 induces the expression of *NtRFP*1, which in turn ubiquitinates βC1 to promote its degradation by the 26S proteasome pathway (Shen et al. 2016). Supporting the evidence, it was also demonstrated that *NtRFP1* overexpression attenuates symptoms exhibited by either βC1 alone or by a virus with functional βC1 and silencing of *NtRFP1* displays enhanced symptom severity in tobacco (Shen et al. 2016). Geminivirus, tomato leaf curl Gujarat virus (ToLCGV) infection in *N. benthamiana* overexpressing tobacco *RDR1* gene (*NtRDR1*) shows symptom recovery, and the plant exhibited reduced expression of *COP9 complex subunit-7* suggesting the involvement of system other than UPS in defense against ToLCGV (Prakash et al. 2020). The RING-finger E3 ubiquitin ligase OsRFPH2-10 plays an antiviral role and mediates the degradation of rice dwarf virus (RDV) encoded P2 protein through proteasome pathways during the early phase of infection (Liu et al. 2014). S-adenosylmethionine (SAM) is a vital methyl donor for several pathways in plants (Wink 1997). Members of SAM pathways also use ubiquitin as a tool of defense against viruses (Mäkinen and De 2019). SAM decarboxylase3 (SAMDC3) from wheat and *N. benthamiana* interact with γb of Barley stripe mosaic virus and positively regulate the 26S proteasomal pathway against the virus. SAMDC3 is shown to ubiquitinate γb in the PEST (Proline, Glutamate, Serine, and Threonine) domain. Further, overexpression of SAMDC3 led to the destabilization of γb and reduced viral infection, while the silencing led to enhanced viral infection (Li et al. 2022).

## Proviral roles of UPS

**Fig. 2:**
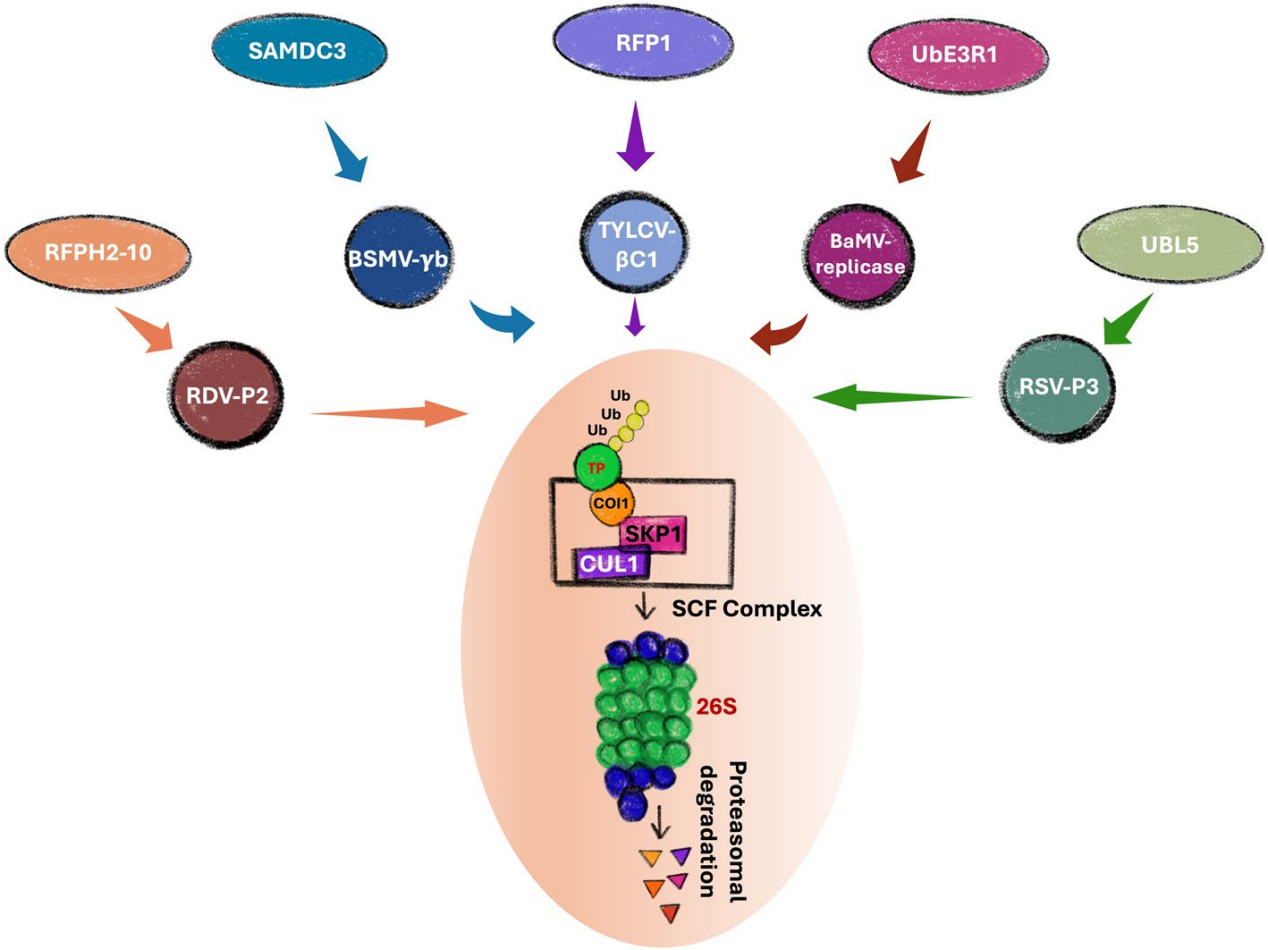
Schematic image depicting host factor promoting viral proteins or the factors towards ubiquitin mediated proteasomal pathways to suppress viral infection. UBL5- ubiquitin like protein 5, RSV- Rice strip virus, NbUbE3R1- Ubiquitin E3 Ligase containing RING domain 1, BaMV- Bamboo mosaic virus, RFP1- RING-finger protein 1, TYLCV- Tomato yellow leaf curl virus, RFPH 2–10- RING-finger E3 ubiquitin ligase, RDV- Rice dwarf virus, SAMDC3- S-adenosyl Methionine decarboxylase 3, and BSMV- Barly strip mosaic virus

Viruses, with their limited coding capacity, encode for fewer multifunctional and specialized proteins to establish infection and counter host defense pathways. One typical example of viruses evolving specialized proteins to counter host defense machinery are VSRs. VSRs have been characterized in all the known viruses and exhibit a vast diversity of functionality to block RNA silencing machinery (Baulcombe 2004; Basu et al. 2014). The evolution of VSRs to counter RNA silencing machinery and manipulation of miRNAs by viruses led to questions about how viruses have evolved measures to counter and even exploit UPS machinery for their benefit. Viruses are known to hijack host cellular machinery for their advantage, and it is interesting to envision how viruses would subvert and exploit different components of UPS machinery in their favor if they hijack host UPS machinery. With the hijacking of host UPS machinery, viruses can neutralize host defense through the degradation of host defense proteins (Fig. 2). In this section, we will emphasize examples of viruses targeting and utilizing host UPS machinery to their advantage.

DNA methylation of the cytosine base is a reversible epigenetic marker that plays a significant role in regulating gene expression, and transposon and transgene silencing (Mahfouz 2010; Wambui Mbichi et al. 2020; Kawakatsu 2020). Plants use DNA methylation to defend against DNA viruses (Raja et al. 2008; Buchmann et al. 2009; Yang et al. 2011; Guo et al. 2022). The major players in the RNA-directed DNA methylation (RdDM) pathway are methyl group donor SAM, domains rearranged methyltransferase2 (DRM2), methyltransferase1 (MET1), Chromomethylase2 (CMT2) and Chromomethylase3 (CMT3). VARIANT IN METHYLATION5 (VIM5) is a ubiquitin E3 ligase that directly targets the DNA methyltransferases, METHYLTRANSFERASE 1 (MET1) and CHROMO-METHYLASE 3 (CMT3) for degradation through the ubiquitin-26S proteasome proteolytic pathway (Mahfouz 2010; Kawakatsu 2020).

Geminivirus, beet severe curly top virus (BSCTV), utilizes VIM5 to reduce symmetric methylation in the promoter region (Chen et al. 2020b). BSCTV infection induces *VIM5* expression in rosette leaf tissues of *Arabidopsis* through replication-initiator protein, which activates the expression of C2 and C3 proteins, leading to reduced symmetric methylation in the promoter of *C2-3* and the onset of disease symptoms (Chen et al. 2020b). The same BSCTV-C2 interacts with S-adenosyl-methionine decarboxylase 1 (SAMDC1) (Zhang et al. 2011), a key enzyme, for the maintenance of S-adenosyl-methionine (SAM)/decarboxylated S-adenosyl-methionine (dcSAM) balance and trans-methylation. BSCTV-C2 hijacks the 26S proteasome pathway to stabilize SAMDC1 by attenuating the degradation of SAMDC1 to counter host DNA methylation-mediated gene silencing of the viral genome (Zhang et al. 2011). Song and coworkers identified a novel major latex protein-like protein 43 in *Nicotiana benthamiana* (NbMLP43) that conferred resistance to potato virus Y (PVY) infection (Song et al. 2023). Interestingly, PVY infection strongly induced *NbMLP43* at the transcription level, but the plants had compromised the NbMLP43 protein level. Upon further investigation, it was observed that PVY uses UPS to degrade NbMLP43 via B-box zinc finger protein 24 (NbBBX24), a light-responsive factor, which is supported by the direct interaction of NbMLP43 with NbBBX24. Ubiquitination occurred at lysine 38 (K38) within NbMLP43, and as a proof of concept, non-ubiquitinated NbMLP43(K38R) conferred stronger resistance to RNA viruses (Song et al. 2023).

The C2 protein from tomato yellow leaf curl Sardinia virus (TYLCSV), tomato yellow leaf curl virus (TYLCV), and beet curly top virus (BCTV) interacts and interferes with the activity of COP9 signalosome (CSN), CSN5 by derubylation of CUL1. Consequently, the interference alters several responses regulated by the CUL1-based SCF ubiquitin E3 ligases, such as abscisic acid, auxins, ethylene, gibberellins, etc., and importantly, the jasmonate response to accelerating the viral infection. Supporting this observation, methyl jasmonate (MeJA) treatment hindered viral infection (Lozano-Durán et al. 2011). A similar observation was made in the case of a Reovirus, rice black-streaked dwarf virus (RBSDV), where the RBSDV encoded P5-1 protein interferes with the CSN-mediated derubylation of OsCUL1 in rice by interacting with OsCSN5A, affecting the JA responsive genes (He et al. 2020). Unlike C2-expressing transgenic *Arabidopsis* plants, P5-1 overexpression in transgenic rice did not overcome the infection upon application of JA (He et al. 2020).

Phytohormones are an integral part of the plant life cycle and regulate a plethora of processes, e.g., seed germination, root and shoot development, reproduction, flowering, biotic and abiotic responses, etc. Phytohormone-mediated defense signaling forms an essential layer of defense against pathogens and activates downstream signaling. These signaling pathways rely on the UPS, specifically E3 Ub ligases, to perceive and initiate signaling transduction. The nexus of phytohormones-UPS mediated defense signaling transduction becomes a crucial target of viruses to neutralize phytohormones-mediated antiviral defense. The Auxin /indole-3-acetic acid proteins (Aux/IAA) family members are short lived nuclear proteins that act as a repressor for auxin signaling, and degradation of Aux/IAA via SKP1-Cullin-F-box^TIR1^ (SCF^TIR1^) is essential for activation of auxin signaling (Abel et al. 1994). The crinivirus, tomato chlorosis virus (ToCV)-p22, can suppress the auxin signaling by hijacking the ubiquitin pathway in plants to promote ToCV infection. The ToCV-encoded p22 protein inhibits auxin signaling by binding to the C-terminal of SKP1 and interferes with the formation of SKP1-Cullin-F-box^TIR1^ (Liu et al. 2021).

NONEXPRESSER OF PATHOGENESIS-RELATED PROTEINS1 (NPR1) functions as the master regulator of systemic acquired resistance mediated by salicylic acid (Cao et al. 1997; Shah et al. 1997; Ryals et al. 1997). A Tenuivirus, rice Stripe Virus (RSV), targets rice NPR1 (OsNPR1) to modulate SA mediated defense signaling. RSV encoded P2 protein promotes degradation of OsNPR1 by enhancing the association of OsNPR1 and the cullin-RING ubiquitin ligases OsCUL3a in a salicylic acid (SA)-independent manner (Zhang et al. 2023). It is important to note that OsNPR1, a master regulator of SA signaling, activates jasmonic acid (JA) signaling, which is crucial for defense against insect vectors (Zhang et al. 2023). The activation of JA signaling by OsNPR1 disrupts the OsJAZ-OsMYC complex and boosts the transcriptional activation activity of OsMYC2 to modulate rice antiviral immunity cooperatively. Interestingly, similar suppression was also observed in unrelated viruses (Zhang et al. 2023). C2 protein of TYLCSV affects downstream signaling of several phytohormones such as that of auxin, gibberellic acid (GA), ethylene (ET), SA and JA by interacting with COP9 signalosome 5 (CSN5) and alters the derubylation activity of the CSN (Lozano-Durán et al. 2011). Studies using transcriptomics and challenge inoculation tools with *A. thaliana* expressing TYLSCV-C2 also support the TYLSCV-C2 mediated suppression of JA signaling mediated defense (Rosas-Díaz et al. 2016).

Plants synthesize JA in response to developmental and environmental stimuli such as necrotrophic pathogen or herbivore attacks (Howe and Jander, 2008; Campos et al., 2014). In plants, JA biosynthesis is repressed by JAZ repressors, and perception of insect pathogens or herbivores induces the synthesis of jasmonoyl-L-isoleucine (JA-Ile). JA-Ile interacts with an E3 ligase named SKP1/CUL1/F-box ^coronatine insensitive1^ (SCF^COI1^) to promote UPS-mediated degradation of JAZ repressors, thereby activating the JA signaling pathway (Ruan et al. 2019). Viruses depend on their insect vector for dispersal inside healthy host plants. Virus interference with JA signaling compromises the immune system in plants and weakens the plant defense against insects in a way that attracts the insect vector to the virus-infected plant. This phenomenon is well documented in begomoviruses, where the virus applies various strategies to suppress JA signaling by modulating the ubiquitin-mediated proteasomal pathway to promote the performance of insect vector or attract the vector toward the infected plants to accelerate the spread of the virus. The βC1 protein encoded by betasatellite molecule cotton leaf curl Multan betasatellite (CLCuMuB) associated with monopartite begomoviruses cotton leaf curl Multan virus (CLCuMuV) interacts with tomato ubiquitin conjugase 3 (SlUBC3) and down-regulates the ubiquitination of proteins as a counter defense. This observation was further supported by the evidence that transgenic tobacco plants over-expressing CLCuMuB-βC1 had reduced ubiquitinated proteins (Eini et al. 2009). Begomoviruses evolved mechanisms to suppress the degradation of JAZ repressor as an efficient strategy to inhibit JA signaling. The CLCuMuB-βC1 interacts with S-phase kinase-associated protein (SKP1) and Cullin 1 (CUL1), essential components of SCF complexes (Jia et al. 2016). CLCuMuB-βC1 also damages the integrity of the SCF^COI1^ complex by interfering with SKP1 and CUL1 to hinder JA responses (Jia et al. 2016). Tomato yellow leaf curl virus (TYLCV) also adapts a similar strategy to subvert plant defense against insect vectors by hijacking the ubiquitin machinery, and it is found to be conserved in plants such as tobacco, tomato, and Arabidopsis. The TYLCV-C2 interacts with RPS27A, essential for the ubiquitin-mediated proteasomal degradation of the repressor JAZ1 (Li et al. 2019). In this case, the expression of JA-responsive genes such as MYC2 (basic helix-loop-helix transcription factor)-regulated genes associated with terpene biosynthesis was observed. Suppression of terpenes attracts the insect vectors towards the infected plants. The same study also reported a similar strategy in another monopartite begomoviruses papaya leaf curl China virus (PaLCuCNV) (Li et al. 2019). RDV encoded Pns11 protein interacts with rice S-adenosyl-L-methionine synthetase (SAMS), which is a key component of the ethylene biosynthesis pathway and enhances its enzymatic activity. This interaction results in increased ethylene production and elevated susceptibility to RDV. Transgenic plants expressing Pns11 or OsSAMS1 had increased levels of RDV while the OsSAMS1 knockout plants resisted RDV infection more efficiently (Zhao et al. 2017).

The HR response is an essential component of plant immunity induced by pathogens-specific signatures and cause rapid cells death at the site of infection thus limiting the infection (Jones and Dangl 2006). In recent years, our understanding of HR regulation by ubiquitin (Ub) and the 26S UPS has grown significantly. Viruses have evolved different strategies to counter HR through controlling UPS machinery. Infection of beet necrotic yellow vein virus (BNYVV) in sugar beets with the *Rz1* resistance gene induces an HR response against BNYVV in resistant varieties. Rz1 overcoming BNYVV isolates can counter this defense through the BNYVV, P25 protein. P25 interacts with sugar beet F-box protein and inhibits the formation of SCF complex to counter host defense response (Thiel and Varrelmann 2009; Thiel et al. 2012). Oat dwarf virus (ODV) belongs to the genus *Mastrevirus* under the family *Geminviridae.* The RepA protein encoded by ODV was shown to induce HR in non-host *Nicotiana benthamiana* (Qian et al. 2016). Analysis of differential expression of genes (DEGs) revealed a complex and dynamic regulatory network involved in modulating RepA-induced HR using transient expression of ODV RepA (Hou et al. 2018). Further investigation revealed that the RepA-induced HR is due to the interaction of RepA with RING-type E3 ligase protein named RING-Finger protein (RFP). The overexpression of *NbRFP1* conferred enhanced resistance against the host, while vice-versa was observed when the gene was downregulated (Liang et al. 2023).

## Conclusion and future perspective

The UPS plays an indispensable role in cellular processes, including defense against invading pathogens. The UPS machinery can intervene at every step of the virus infection cycle by targeting viral proteins for degradation. Viruses, in turn, have evolved specialized mechanisms to counter UPS machinery and even exploit UPS components for their advantage. Most studies conducted focused on the UPS -virus interactions with one virus but in nature the same host is infected by multiple viruses at the same time, it would be interesting to study the interaction between the UPS system and multiple viruses in synergistic and antagonistic virus infections. These studies could be beneficial to develop durable and broad spectrum antiviral strategies. The dynamic interactions between viruses and the UPS system occur at the protein level and requires sensitive techniques to uncover this interaction. Due to the weak interactions between E3 ligases and their known substrates and the rapid degradation of the target protein, it is challenging to capture the comprehensive interactome during UPS-virus interactions. Traditional molecular biology tools used to study UPS-virus interactions include ubiquitin-specific antibodies, affinity purification coupled with mass spectrometry (AP-MS), yeast two-hybrid screens, use of proteasome inhibitor drugs such as MG132.. To this end, new sensitive technologies are required, both in vitro and in vivo, that can contribute to the discovery and characterization of still-unknown substrates. The comprehensive understanding of the UPS-virus interactions will facilitate the development of virus-resistant plants using the modern biotechnological tools such as CRISPR-Cas9 mediated genome editing of host susceptibility factor.

## Data Availability

Not applicable.

## Abbreviations

UPS: Ubiquitin-proteasome systems
PTI: Pattern-triggered immunity
ETI: Effector-triggered immunity
HR: Hypersensitive response
TMV: Tobacco mosaic virus
TYMV: Turnip yellow mosaic virus
RdRp: RNA-dependent RNA polymerase
PLRV: Potato leafroll virus
MP: Movement protein
PVX: Potato virus X
ERAD: ER-associated protein degradation
TBSV: Tomato bushy stunt virus
RSV: Rice stripe virus
BaMV: Bamboo mosaic virus
TRV: Tobacco rattle virus
TYLCV: Tomato yellow leaf curl virus
VIGS: Virus induced gene silencing
ToLCGV: Tomato leaf curl Gujarat virus
RDV: Rice dwarf virus
SAM: S-adenosylmethionine
SAMDC3: SAM decarboxylase3
VSRs: Viral suppressors of gene silencing
RdDM: RNA-directed DNA methylation
DRM2: Domains rearranged methyltransferase2
MET1: Methyltransferase1
VIM5: VARIANT IN METHYLATION5
BSCTV: Beet severe curly top virus
SAMDC1: S-adenosyl-methionine decarboxylase1
PVY: Potato virus Y
TYLCSV: Tomato yellow leaf curl Sardinia virus
BCTV: Beet curly top virus
CSN: COP9 signalosome
RBSDV: Rice black-streaked dwarf virus
ToCV: Tomato chlorosis virus
NPR1: NONEXPRESSER OF PATHOGENESIS-RELATED PROTEINS1
RSV: Rice Stripe Virus
JA: Jasmonic acid
JA-Ile: Jasmonoyl-L-isoleucine
CLCuMuB: Cotton leaf curl Multan betasatellite
CLCuMuV: Cotton leaf curl Multan virus
PaLCuCNV: Papaya leaf curl China virus
SAMS: S-adenosyl-L-methionine synthetase
BNYVV: Beet necrotic yellow vein virus
ODV: Oat dwarf virus
PL: Proximity labeling
